# IVIg Immune Reconstitution Treatment Alleviates the State of Persistent Immune Activation and Suppressed CD4 T Cell Counts in CVID

**DOI:** 10.1371/journal.pone.0075199

**Published:** 2013-10-09

**Authors:** Dominic Paquin-Proulx, Bianca A. N. Santos, Karina I. Carvalho, Myrthes Toledo-Barros, Ana Karolina Barreto de Oliveira, Cristina M. Kokron, Jorge Kalil, Markus Moll, Esper G. Kallas, Johan K. Sandberg

**Affiliations:** 1 Center for Infectious Medicine, Department of Medicine, Karolinska Institutet, Karolinska University Hospital Huddinge, Stockholm, Sweden; 2 Division of Clinical Immunology and Allergy, University of Sao Paulo School of Medicine, Sao Paulo, Brazil; New York University, United States of America

## Abstract

Common variable immunodeficiency (CVID) is characterized by defective B cell function, impaired antibody production, and increased susceptibility to bacterial infections. Here, we addressed the hypothesis that poor antibody-mediated immune control of infections may result in substantial perturbations in the T cell compartment. Newly diagnosed CVID patients were sampled before, and 6–12 months after, initiation of intravenous immunoglobulin (IVIg) therapy. Treatment-naïve CVID patients displayed suppressed CD4 T cell counts and myeloid dendritic cell (mDC) levels, as well as high levels of immune activation in CD8 T cells, CD4 T cells, and invariant natural killer T (iNKT) cells. Expression of co-stimulatory receptors CD80 and CD83 was elevated in mDCs and correlated with T cell activation. Levels of both FoxP3+ T regulatory (Treg) cells and iNKT cells were low, whereas soluble CD14 (sCD14), indicative of monocyte activation, was elevated. Importantly, immune reconstitution treatment with IVIg partially restored the CD4 T cell and mDC compartments. Treatment furthermore reduced the levels of CD8 T cell activation and mDC activation, whereas levels of Treg cells and iNKT cells remained low. Thus, primary deficiency in humoral immunity with impaired control of microbial infections is associated with significant pathological changes in cell-mediated immunity. Furthermore, therapeutic enhancement of humoral immunity with IVIg infusions alleviates several of these defects, indicating a relationship between poor antibody-mediated immune control of infections and the occurrence of abnormalities in the T cell and mDC compartments. These findings help our understanding of the immunopathogenesis of primary immunodeficiency, as well as acquired immunodeficiency caused by HIV-1 infection.

## Introduction

Common variable immunodeficiency (CVID) is one of the most common primary immune deficiency and is characterized by low levels of IgG and IgA [1], [2]. Several genetic mutations associated with CVID have been identified, but in many cases the exact cause is unknown [2]. CVID patients thus represent a heterogeneous group, sharing a phenotype with impaired B cell function. This results in poor humoral immunity and recurrent bacterial infections, primarily of the upper respiratory and gastrointestinal tracts [3].

The treatment for CVID is IgG replacement, often given as intravenous immunoglobulins (IVIg), consisting of monomeric IgG purified from pooled plasma from healthy donors [3]. IVIg acts mainly as a reconstitution therapy, providing patients with pathogen-specific antibodies and protection from infections. After IVIg initiation, patients usually experience significant improvement in their quality of life with reduced rate and severity of infections and fewer days of hospitalization. Efficiency of IVIg treatment in CVID patient has been associated with polymorphism of the neonatal Fc receptor [4]. In addition to its use in CVID, IVIg is also used to treat an increasing number of autoimmune and inflammatory diseases. In such diseases, the mechanisms of action of IVIg are complex and the Fc region, the Fab region, the complement binding regions as well as sialic acid are all proposed to be involved [5]. Similarly, IVIg may play diverse roles in treatment of immune deficiencies beyond being solely reconstitution therapy [6].

In contrast to the defects in humoral immunity, T cell-mediated control of viral infections is believed to be mostly preserved in CVID patients, although an inverted CD4/8 ratio is often observed. However, recent studies have indicated that CVID patients on IVIg treatment exhibit signs of systemic immune activation [7], [8]. This type of immune activation shares characteristics with that observed in secondary immunodeficiency caused by HIV-1 infection. Chronic pathological immune activation contributes strongly to the progression of HIV-1 disease [9], [10], [11], [12], [13], [14], and possible approaches to control immune activation using various forms of immunotherapy are therefore of great interest.

In the present study, we hypothesized that poor antibody-mediated immune control of bacterial infections in untreated CVID patients might result in considerable perturbations of the T cell and the myeloid dendritic cell (mDC) compartment. We found that treatment-naïve CVID patients had severely suppressed CD4 T cell counts, as well as low levels of invariant natural killer T (iNKT) cells and FoxP3+ T regulatory (Treg) cells, consistent with previous reports. This was paired with high levels of T cell activation and exhaustion, altered expression of co-stimulatory receptors in mDCs, and elevated levels of sCD14 in plasma. Interestingly, immune reconstitution treatment with IVIg partially restored the CD4 T cell compartment and reduced CD8 T cell activation. These findings demonstrate that significant perturbations occur in the T cell compartment in CVID, and that these are partially reversed by IVIg treatment. We discuss these findings in CVID in the context of the similarities that exist with markers of the immunopathogenic process in HIV-1 disease.

## Materials and Methods

### Study cohort and samples

CVID patients (aged 22–59) and healthy controls (aged 21–66) were enrolled at the University of Sao Paulo (USP) (Table 1). None of the patients suffered from active infection at the time of enrollment. The study was approved by the USP institutional review board, and written informed consent was provided by all participants according to the Declaration of Helsinki. Peripheral blood mononuclear cells (PBMC) were isolated by density-gradient sedimentation using Ficoll-Paque (Lymphoprep, Nycomed Pharma, Oslo, Norway). Isolated PBMC were washed twice in Hank's balanced salt solution (Gibco, Grand Island, NY), and cryopreserved in RPMI 1640 (Gibco), supplemented with 20% heat inactivated fetal bovine serum (FBS; Hyclone Laboratories, Logan UT), 50 U/ml of penicillin (Gibco), 50 µg/ml of streptomycin (Gibco), 10 mM glutamine (Gibco) and 7.5% dimethylsulphoxide (DMSO; Sigma, St Louis, MO). Cryopreserved cells from all subjects were stored in liquid nitrogen until used in the assays. For CVID patients, samples were collected before, and 6–12 months after, initiation of IVIg treatment.

**Table 1:** Characteristics of CVID patients.

Subject	Gender	Age	Baseline IgG	Baseline IgA	CD4 count	CD8 count	CD4/CD8	B cell count	Autoimmunity
			(mg/dl)	(mg/ml)	(cell/µl)	(cell/µl)	ratio	(cell/µl)	
CVID-1	F	59	10	4	489	1749	0.28	58	no
CVID-2	M	27	100	4	362	206	1.76	188	no
CVID-3	F	57	73	10	224	579	0.39	245	no
CVID-4	M	32	10	4	283	1182	0.24	0	no
CVID-5	M	22	16	4	548	1834	0.3	NA	no
CVID-6	F	29	10	4	259	472	0.55	120	no
CVID-7	F	57	38	4	250	406	0.62	55	no
CVID-8	M	22	27	4	533	678	0.79	253	no
CVID-9	M	40	23	4	766	1233	0.62	22	no
CVID-10	M	27	117	NA	154	254	0.61	NA	no
CVID-11	F	30	388	3	470	574	0.82	122	ITP
CVID-12	F	28	118	4	339	582	0.58	218	no

### Clinical immunology

Peripheral blood absolute T cell counts were assessed using the BD Tritest anti-CD4-FITC/anti-CD8-PE/anti-CD3-PerCP mAb cocktail and BD TruCount Tubes (BD Biosciences, San Diego, CA), according to the manufacturer's instruction, using a FACSCalibur flow cytometer (BD Biosciences).

### Flow cytometry and mAbs

Cryopreserved specimens were thawed and washed, and counts and viability were assessed using the Countless Automated Cell Counter system (Invitrogen, Carlsbad, CA). Cells were washed and stained at room temperature for 10 min in 96-well V-bottom plates in the dark. Samples were then washed and fixed using 2% formaldehyde before flow cytometry data acquisition. mAbs used in flow cytometry; anti-CD3 AF700, anti-CD3 V450, anti-CD4 APC-H7, anti-CD11c APC-Cy7, anti-CD25 PE-Cy7, anti-CD38 PE-Cy7, anti-CD40 FITC, anti-CD56 AF700, anti-CD80 PE, anti-CD83 PE-Cy7, anti-CD86 v450, anti-CD152 (CTLA4) PE, anti-CD161 APC, anti-CD279 (PD-1) PE, anti-HLA-DR APC, anti-HLA-DR PE-Cy7 were all from BD Biosciences (San Jose, CA), anti-CD3 eF450 was from eBiosciences (San Diego, CA), anti-CD8 PE-Texas Red and anti-CD19 PE-Texas Red were from Abcam (Cambridge, UK), anti-CD14 PE-Texas Red and anti-HLA-DR PE-Texas Red were from Invitrogen, anti-CD279 PB was from Biolegend (San Diego, CA), anti-Vα24 FITC and anti-Vβ11 PE were from Beckman Coulter (Fullerton, CA). For staining of FoxP3 and Ki67, samples were first stained for extracellular markers as described above, and then washed, permeabilized and fixed using a FoxP3 staining kit (eBioscience), and stained with anti-FoxP3 APC (BioLegend) or anti-Ki67 FITC (BD Biosciences). Flow cytometry data was acquired using a BD LSRFortessa instrument (BD Biosciences) and analyzed using FlowJo version 9.3 (Tree Star, Ashland, OR).

### sCD14 measurement

sCD14 was measured in plasma by ELISA following manufacturer's instruction (R&D Systems, Minneapolis, MN).

### Statistical analysis

All statistical analysis was performed using Graph Pad Prism version 5.0a for Mac OSX (GraphPad Software, La Jolla, CA). The comparison between healthy controls and CVID patients was analyzed using Mann Whitney test and changes after IVIg initiation in CVID patients were analyzed with Wilcoxon marched-pairs signed rank test. Associations between groups were determined by Spearman's rank correlation. P values<0.05 were considered statistically significant.

## Results

### Suppressed CD4 T cell counts in CVID patients are partially restored by IVIg treatment

**Figure 1:**
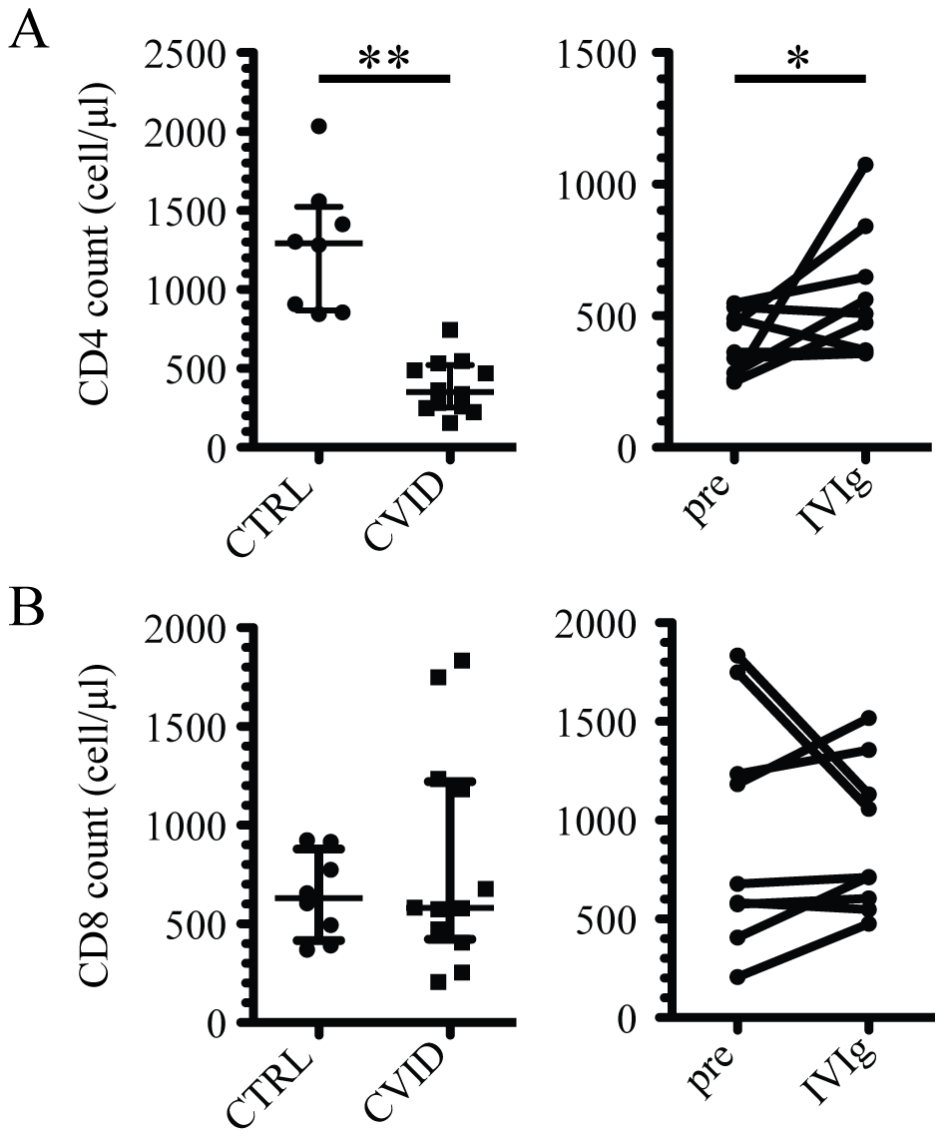
CD4 T cells, but not CD8 T cells, are reduced in CVID patients and partially recover after IVIg treatment. (A) CD4 T cell counts in healthy control subjects and CVID patients before initiation of IVIg (left panel), and comparison of CD4 T cell counts in CVID patients before and after IVIg (right panel). (B) CD8 T cell counts in healthy control subjects and CVID patients before initiation of IVIg (left panel), and comparison of CD8 T cell counts in CVID patients before and after IVIg (right panel). ** indicates p<0.001 and * indicates p<0.05.

Twelve patients newly diagnosed with CVID according to the criteria established by the Pan-American Group for Immunodeficiency, and thirteen gender and age matched healthy controls, all HIV-negative, were enrolled in the study (Table 1). CD4 T cell counts were significantly reduced in CVID patients as compared with the healthy control group (p<0.001) (Figure 1A, left panel), whereas CD8 T cell counts were similar between the groups (Figure 1B, left panel), although a few CVID patients exhibited relatively high CD8 cell counts. Immune reconstitution treatment with IVIg was started soon after CVID diagnosis according to standard clinical protocols. Overall, six of the nine patients for whom cell counts were available showed increased CD4 T cell counts 6–12 months after initiation of IVIg (p<0.05) (Figure 1A, right panel). In contrast, CD8 T cell counts were unchanged by IVIg treatment (Figure 1B, right panel). Thus, the low CD4 T cell counts in untreated CVID patients partially recover after immune reconstitution with IVIg.

### High levels of T cell activation in CVID are alleviated by immune reconstitution treatment

**Figure 2:**
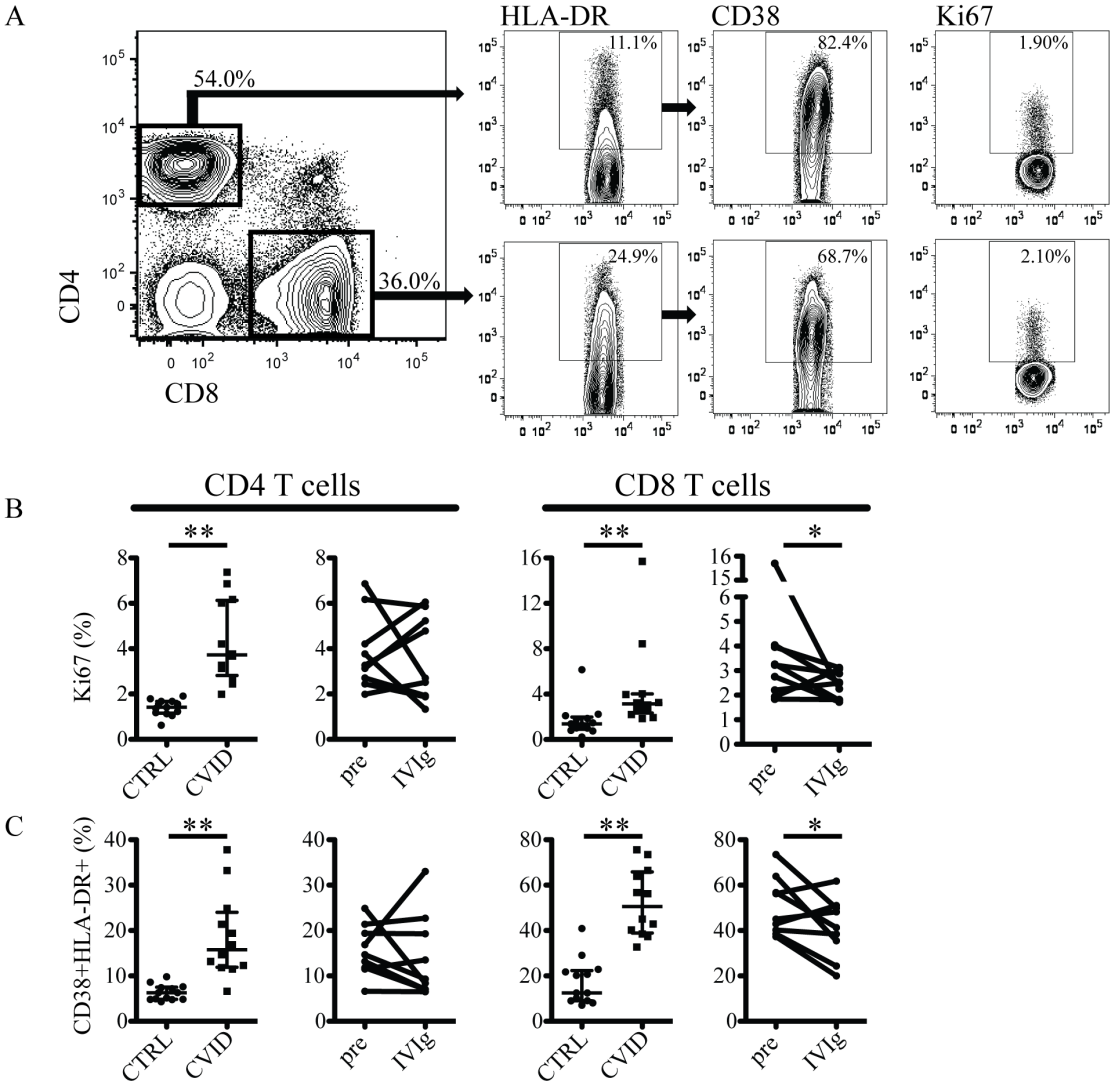
Immune activation in CD4 and CD8 T cells in CVID patients and the effect of immune reconstitution treatment. (A) Representative staining illustrating the gating strategy used. (B) Comparison of the levels of Ki67 expression in CD4 and CD8 T cells between healthy control and CVID patients (left panel), and in CVID patients before and after IVIg (right panel). (C) Levels of CD38+HLA-DR+ cells in CD4 and CD8 T cells in healthy control and CVID patients (left panel), and in CVID patients before and after IVIg (right panel). ** indicates p<0.002 and * indicates p<0.05.

With the data on low CD4 T cell counts in CVID patients at hand, we next examined the level of immune activation in the CD4 and CD8 T cell compartments of CVID patients (Figure 2A). The frequency of cycling cells, expressing Ki67, was elevated in both CD4 and CD8 T cells of CVID patients as compared to controls (Figure 2B). Following IVIg therapy, Ki67 expression decreased in CD8 T cells in six out of nine patients, whereas no change occurred in the CD4 T cell compartment (Figure 2B). T cell activation, measured as expression of HLA-DR (Figure S1A), and co-expression of CD38 and HLA-DR (Figure 2C), was high in both CD4 and CD8 T cells. In addition, the intensity of CD38 expression (mean fluorescence intensity, MFI) was elevated on CD8 but not on CD4 T cells (Figure S1B). Interestingly, CD38 intensity on CD8 T cells as well as the frequency of CD38+HLA-DR+ CD8 cells were reduced following IVIg (Figure S1B and Figure 2C). Overall, these findings indicate that CVID patients have high activation levels in both CD4 and CD8 T cell compartments, and that activation levels go down in CD8 T cells, but not CD4 T cells, following immune reconstitution treatment. This suggests that reconstitution of humoral immunity may help control infections or infection-associated factors that contribute to immune activation in the CD8 T cell compartment.

### The elevated levels of CD4 T cell PD-1 expression in CVID correlate with age

**Figure 3:**
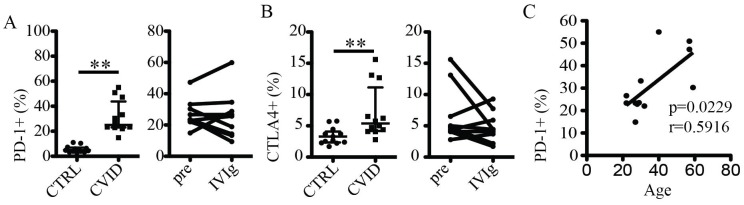
Increased expression of inhibitory receptors on CD4 T cells in CVID. (A) Levels of PD-1 expressed in CD4 T cells in healthy controls and CVID patients (left panel), and in CVID patients before and after IVIg treatment (right panel). (B) Levels of CTLA-4 expressed in CD4 T cells in healthy controls and CVID patients (left panel), and in CVID patients before and after IVIg treatment (right panel). (C) Correlation between patient age and levels of PD-1 expression in CD4 T cells before initiation of IVIg treatment. ** indicates p<0.003.

Programmed Death 1 (PD-1) (CD279) and cytotoxic T lymphocyte antigen 4 (CTLA4) (CD152) are inhibitory receptors and markers of immune cell exhaustion, and high levels of PD-1 and CTLA4 are often observed in T cells in patients suffering from chronic infections [15], [16]. Here, CVID patients had higher levels of PD-1 and CTLA4 on CD4 T cells as compared to healthy controls (Figures 3A and B). However, treatment with IVIg had no measurable effect on PD-1 and CTLA4 expression on T cells over the course of the study (Figures 3A and B).

We next investigated possible associations between T cell activation in treatment-naïve patients and various patient characteristics. No correlation was observed between immune activation parameters and CD4 count or IgG levels before initiation of IVIg (data not shown). However, the frequency of PD-1+ cells in the CD4 T cell compartment (p = 0.023, r = 0.5916), as well as the frequency of HLA-DR+ cells in the CD8 T cell compartment (p = 0.049, r = 0.5026), correlated positively with age (Figure 3C and Figure S1C). These associations were not present in the healthy control group (data not shown). Although cross-sectional in nature, these data may suggest that T cell immune activation and exhaustion in CVID may develop progressively and associated with immune system aging.

### Association between expression of co-stimulatory molecules in mDCs and T cell activation

**Figure 4:**
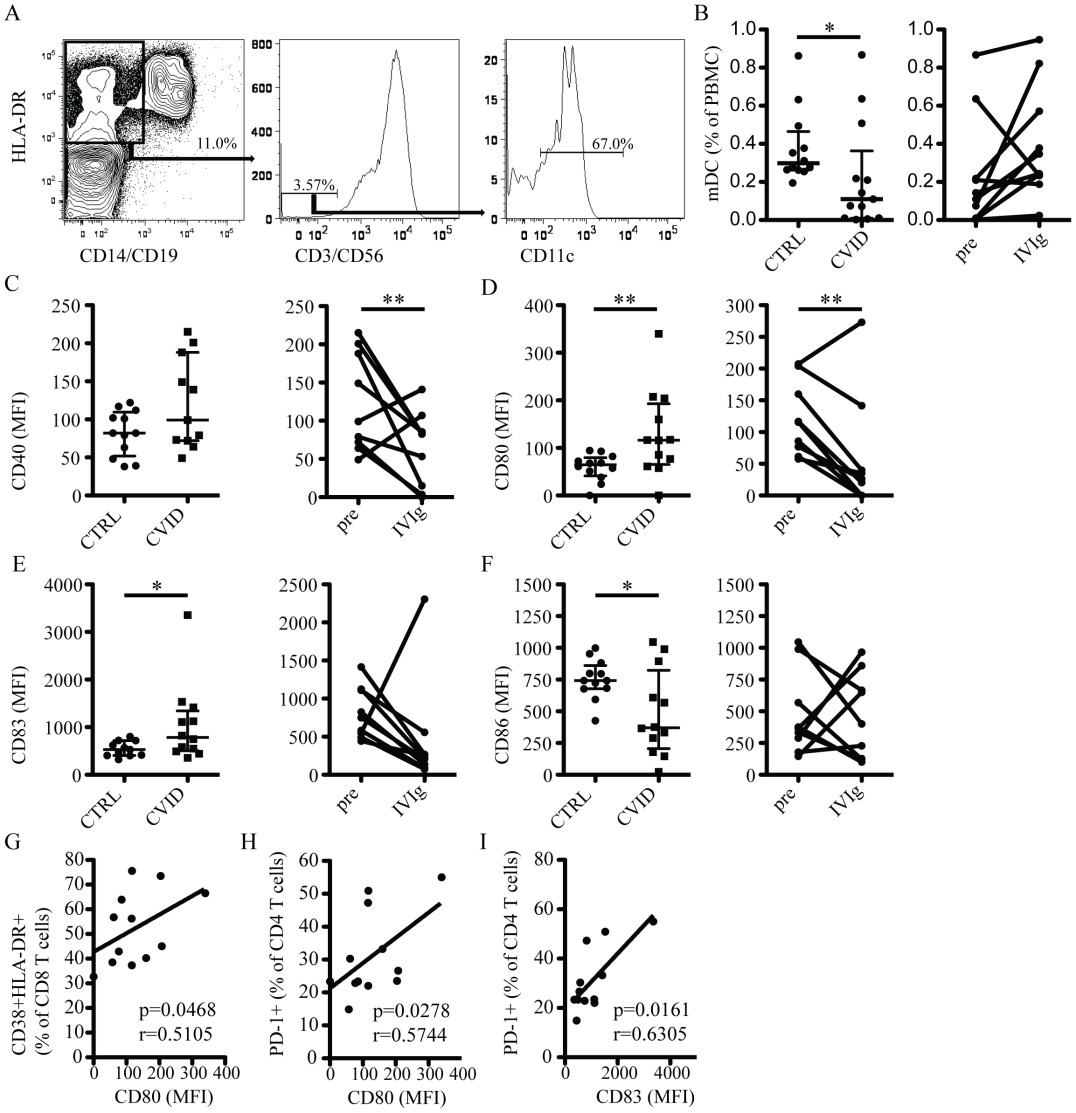
Altered expression of co-stimulatory molecules on mDCs in CVID. (A) Representative staining illustrating the gating strategy used. (B) Frequency of mDCs in blood from healthy controls and CVID patients (left panel), and in CVID patients before and after IVIg treatment (right panel). Comparison of the MFI of CD40 (C), CD80 (D), CD83 (E) and CD86 (F) in healthy controls and CVID patients before IVIg (left panel), and in CVID patients before and after IVIg (right panel). (G) Correlation between CD80 MFI and levels of CD38+HLA-DR+ CD8 T cells. (H) Correlation between CD80 MFI and levels of PD-1+ CD4 T cells. (I) Correlation between CD83 MFI and PD-1+ CD4 T cells. ** indicates p<0.02 and * indicates p<0.05.

DCs are specialized in capturing, processing and presenting antigen to activate the immune system in response to infections. Upon TLR signaling, immature DCs will up-regulate co-stimulatory molecules that provide the second signal to activate T cells. We hypothesized that recurrent bacterial infections in CVID patients might result in increased expression of co-stimulatory molecules on mDC, and that this could be associated with the high levels of T cell activation observed in these patients. mDCs, defined as CD3-CD14-CD19-CD56-CD11c+HLA-DR+ cells (Figure 4A), displayed a sharply reduced frequency in blood from treatment-naïve CVID patients (Figure 4B). Patients with sub-normal levels of mDCs experienced a recovery of these cells on IVIg treatment, and for the whole group this was close to statistically significant (p = 0.05) (Figure 4B). mDCs in patients had similar levels of CD40 as the healthy control subjects (Figure 4C). However, the levels of CD80 and CD83 were higher (Figures 4D and E), and levels of CD86 was lower (Figure 4F), in mDCs from CVID patients as compared to mDCs from healthy controls. Interestingly, the levels of CD40 and CD80 were significantly reduced after reconstitution therapy (p = 0.01 and p = 0.02, respectively), and a similar trend was also observed for CD83 (p = 0.065), while CD86 expression was unchanged. A few studies have investigated the effects of IVIg on the levels of co-stimulatory molecules on DCs *in vitro* and contradicting results were reported [17], [18]. Our results indicate that mDCs from treatment-naïve CVID patients *ex vivo* have an altered expression profile of co-stimulatory molecules, and that IVIg immune reconstitution can partially restore this to normal.

We next evaluated possible associations between expression levels of CD80 and CD83, which were elevated in mDCs from CVID patients, and levels of T cell activation. Interestingly, levels of CD80 on mDC were positively associated with co-expression of CD38 and HLA-DR in CD8 T cells (Figure 4G). Similarly, expression of both CD80 and CD83 on mDCs correlated positively with PD-1 expression on CD4 T cells (Figures 4H and I). These associations are consistent with a model where innate activation of mDCs plays a role in the state of high T cell immune activation in CVID.

**Figure 5:**
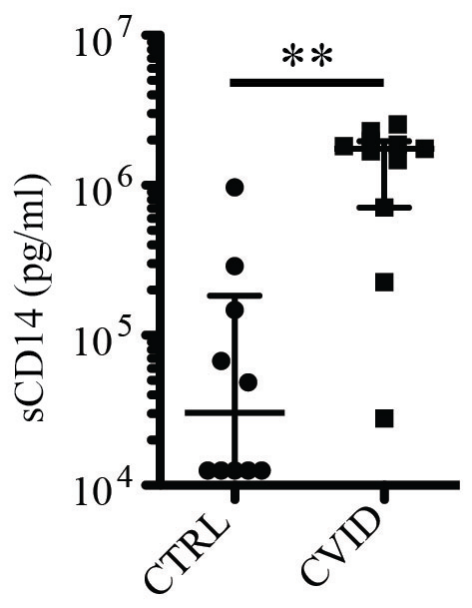
Elevated levels of sCD14 in CVID. Comparison of plasma levels of sCD14 in healthy controls and CVID patients before IVIg treatment. ** indicates p = 0.001.

Soluble CD14 (sCD14) is a plasma marker of innate activation of monocytes and other CD14-expressing cells, and has been studied in particular in HIV-1 infection as a possible marker of microbial translocation over the intestinal barrier [19], [20], [21]. To evaluate the possibility that microbial translocation might occur in CVID patients we measured sCD14 in plasma before IVIg treatment. Treatment-naïve CVID patients had significantly elevated levels of sCD14 in plasma as compared to healthy controls (Figure 5). It is noteworthy that low levels of IgA, like those seen in CVID, have been suggested to contribute to increased microbial translocation [22]. These data support the possibility that microbial translocation might contribute to the elevated levels of innate and adaptive immune activation seen here in CVID.

### iNKT cells and Treg cells are low in CVID and do not recover during IVIg treatment

**Figure 6:**
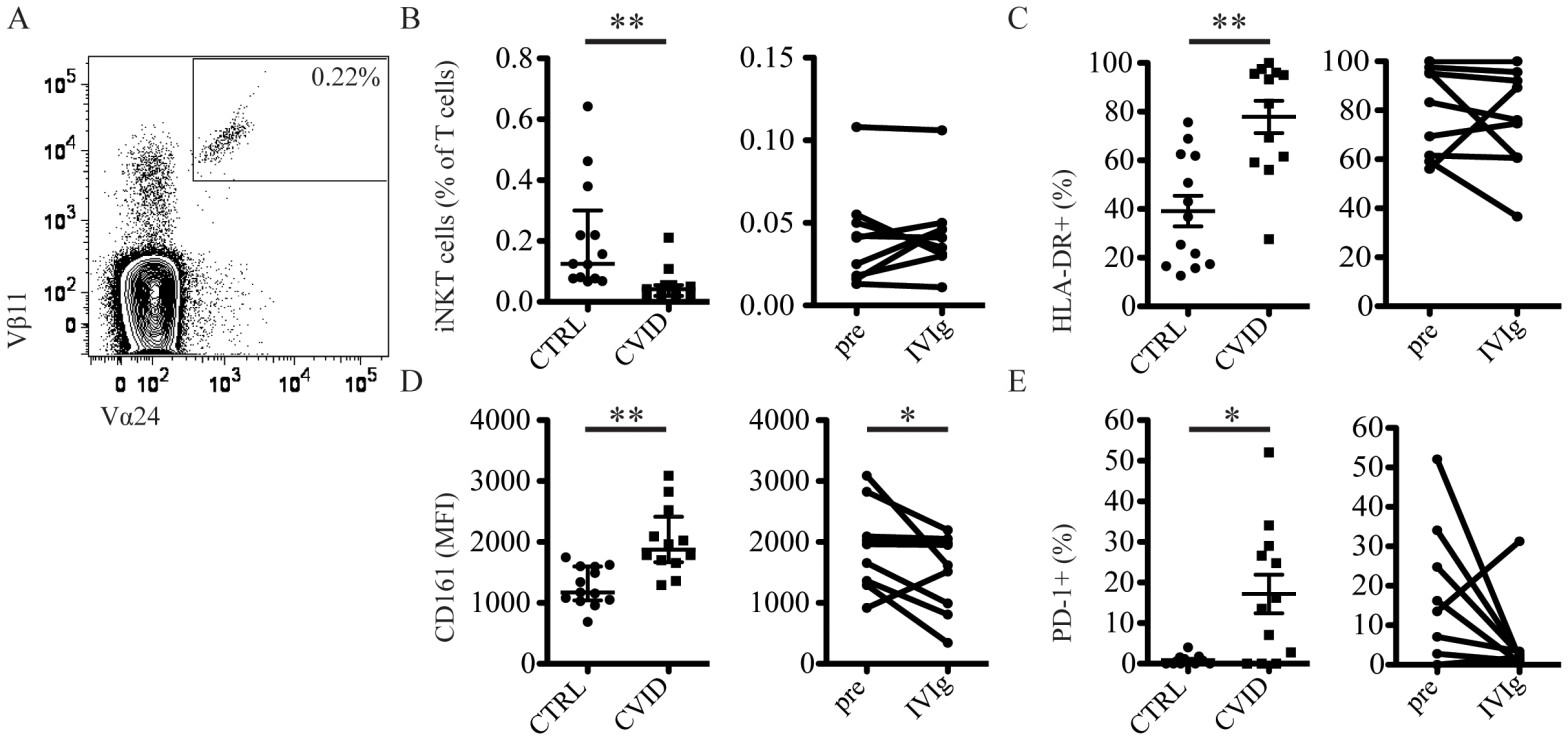
iNKT cells are reduced in CVID. (A) Representative staining illustrating the gating strategy used. (B) Comparison between the frequency of iNKT cells in healthy control subjects and CVID patients before IVIg (left panel), and in CVID patients before and after IVIg (right panel). (C) Expression of HLA-DR on iNKT cells in healthy control and CVID patients before IVIg (left panel), and in CVID patients before and after IVIg (right panel). (D) Expression of CD161 on iNKT cells in healthy control subjects and CVID patients before IVIg (left panel), and in CVID patients before and after IVIg (right panel). (E) Expression of PD-1 on iNKT cells in healthy control subjects and CVID patients before IVIg (left panel), and in CVID patients before and after IVIg (right panel). ** indicates p<0.001 and * indicates p<0.02.

iNKT cells operate on the border between innate and adaptive immunity and recognize endogenous and bacterial glycolipids presented by CD1d [23], [24]. They are believed to be important for immune control of both bacterial and viral infections. iNKT cell levels were reduced by almost five-fold in CVID patients compared to healthy controls, as previously reported [25], and did not recover after IVIg therapy (Figure 6B). Levels of HLA-DR, CD161 and PD-1 (Figures 6C, D, and E, respectively), were elevated on iNKT cells in CVID patients, suggesting ongoing activation and exhaustion in this compartment. IVIg therapy reversed the level of CD161 back to that observed in healthy controls, and a similar trend was observed for PD-1. Similar to the CD4 and CD8 T cell compartments, HLA-DR expression on iNKT cells was not normalized by IVIg treatment.

**Figure 7:**
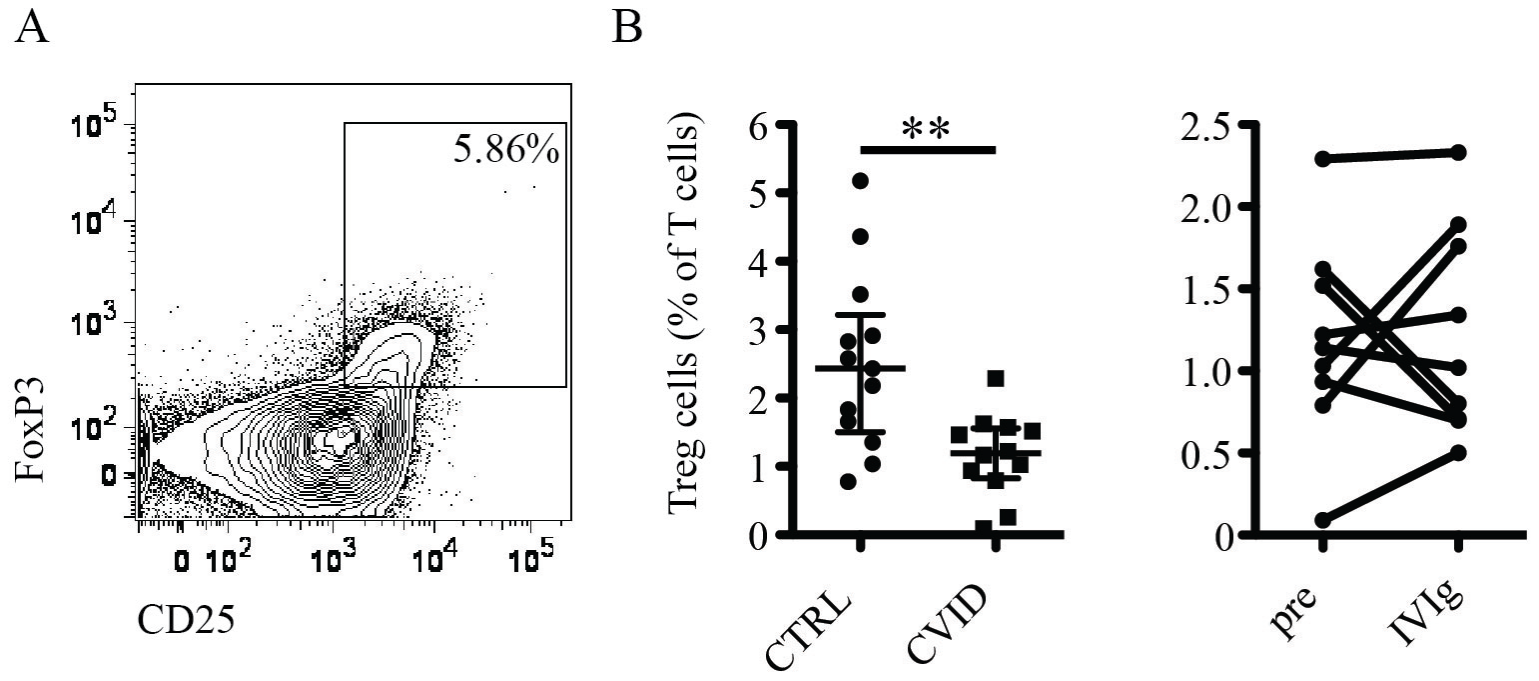
FoxP3+ Treg cells are reduced in CVID. (A) Representative staining illustrating the gating strategy used. (B) Comparison of the frequency of Treg cells in healthy controls and CVID patients before IVIg (left panel), and in CVID patients before and after IVIg (right panel). ** indicates p = 0.002.

Treg cells are important to control immune responses and limit persistent immune activation [26]. We therefore investigated the frequency and phenotype of Treg cells in CVID patients compared to healthy controls. Treg cells were defined as CD4+CD25^hi^FoxP3+ T cells (Figure 7A). Treg cell levels were reduced in CVID patients [27], [28], and did not improve following IVIg treatment (Figure 7B). A previous study found Treg cells to be increased 30 min following IVIg infusion [29]. The present results suggest that such an effect may be transient, as samples obtained after consecutive IVIg treatments showed no consistent effects on the levels of Treg cells.

## Discussion

CVID is associated with defective B cell responses and the concomitant poor antibody-mediated control of microbial infections. In this study, we report substantial perturbations in cell-mediated immunity and high levels of immune activation in newly diagnosed CVID patients sampled before initiation of IVIg treatment. The CD4 T cell compartment was severely affected with suppressed absolute CD4 counts, high activation levels as measured by HLA-DR and CD38 co-expression and Ki67 expression, and immune exhaustion as assessed by expression of PD-1 and CTLA-4. Peripheral blood mDCs were numerically suppressed and displayed an activated phenotype with elevated levels of CD80 and CD83. Interestingly, PD-1 in CD4 T cells correlated with expression levels of these co-stimulatory molecules, suggesting a possible link between innate immune activation in response to microbes and the persistent CD4 T cell activation. The existence of innate immune activation was also supported by the elevated levels of sCD14 in plasma, as well as the high levels of activation in the residual innate-like iNKT cells. Whereas CD8 T cell counts remained similar in patients and controls, these cells were highly activated in CVID and this activation correlated with the expression of CD80 in mDCs. Altogether, the T cell compartment in untreated CVID patients is highly activated, suffer loss of CD4 T cells and mDCs as well as the immunoregulatory Treg and iNKT cells, and these changes are paired and at least to some extent correlated with activation in the innate immune system.

Several of these cellular immunological abnormalities show a partial normalization after humoral immunodeficiency is alleviated with IVIg. This effect is particularly clear in the case of CD4 T cell recovery, the decline in CD8 T cell activation levels, and decline in CD80 levels on mDCs. As the primary therapeutic effect of IVIg in this situation is to provide pathogen-specific antibodies, it is likely that improved immune control of infections is the underlying cause of normalization of these cellular immune parameters. In this model, a reduced microbial pressure on innate and cellular immunity supports a lowering of immune activation and recovery of CD4 T cell counts. This is also consistent with the trend towards recovery of mDCs on treatment. One should also note that iNKT cells and Treg cells do not recover numerically after IVIg treatment, indicating that some pathological changes in the T cell compartment persist despite immune reconstitution treatment.

Many of the pathological changes we have observed in the cellular immune system in CVID bear a resemblance to those commonly seen in chronic untreated HIV-1 infection. This includes low CD4 T cell counts, CD4 T cell expression of markers associated with activation and exhaustion [14], [30], as well as CD8 T cell activation [9], [10], [11], [12], [13], [14]. Likewise, the patterns of decreased levels of mDCs [31], [32], [33], Treg cells [34], [35] and iNKT cells [36], [37], [38], increased PD-1 expression on iNKT cells [39], as well as increased plasma levels of sCD14 [14], [40], in CVID patients are shared with HIV-1 infected patients. Interestingly, it was previously reported that CVID patients with lower naïve CD4 T cell levels display worse clinical scores [41], and, in line with the present data, that CVID patients display a pattern of L-selectin and CD38 expression on CD8 T cells similar to that observed in HIV-1 infected patients [42].

For some of the common immunological features of HIV-1 infection and CVID, most notably the low CD4 T cell counts and CD8 T cell activation, partial normalization was observed in the CVID patients after immune reconstitution treatment with IVIg. Previous studies have reported increased CD4 counts [43], [44], and transient decreases in CD4 and CD8 T cell activation [44], in antiretroviral treatment (ART)-naïve HIV-1 infected patients following infusion of IVIg. We speculate that IVIg could be useful as a supplement therapy for patients that have residual immune activation despite successful virological response to ART. IVIg might in this situation contribute to control of bacterial infections and microbial translocation, and possibly support normalization in immune activation levels and CD4 counts similar to what we have observed here in CVID.

Mice that lack IgA display an up-regulation of innate immune genes in the intestinal epithelium, and similar changes in gene expression were observed in the intestinal epithelium of both CVID patients and HIV-1 infected patients [45]. Events at mucosal barriers may thus be involved in the common features observed between patients with CVID and HIV-1 infected subjects. Our data indicate that PD-1 expression in CD4 T cells as well as activation levels in CD8 T cells in CVID are associated with levels of CD80 and CD83 expression in peripheral blood mDCs. Furthermore, sCD14 is increased in untreated CVID patients. Interestingly, a recent study correlated elevated levels of sCD14 with CD4 T cell loss in CVID patients on stable IVIg treatment, despite no detectable elevation in LPS [8]. These findings together support a model where poor control of infections, and possibly also microbial translocation, due to impaired humoral immunity contribute to chronic immune activation and pathological changes in the cellular immune system that bear similarity with untreated HIV-1 infection. Whereas viral infections may contribute to immune activation in CVID, bacterial and fungal pathogens may be more likely as the dominant drivers of pathological changes in the cell-mediated immune defenses. Interestingly, a recent study by Young et al. based on a murine model indicated that absence of antibodies is associated with activation of endogenous retroviruses (ERVs) [46]. Furthermore, ERV activation in this situation appeared to depend on the intestinal microbiota. It would be very interesting to explore the possible involvement of ERVs in the immunopathological changes and immune activation seen in CVID.

Taken together, we believe that the results presented here are important for the understanding of immunopathogenic processes in CVID, and of the common features that exist between primary and acquired immunodeficiency. The results also provide clues as to the mechanisms that may lead to pathological persistent immune activation, and the downstream effects of this on the human immune system.

## Supporting Information

Figure S1 **Immune activation in CD4 and CD8 T cells in CVID patients and the effect of immune reconstitution treatment.** Comparison of the levels of HLA-DR+ cells (A), CD38 MFI (B), between healthy control and CVID patients (left panel), and in CVID patients before and after IVIg (right panel) for CD4 and CD8 T cells. (C) Correlation between age of the patients at baseline and the levels of HLA-DR expressing CD8 T cells. ** indicates p<0.002 and * indicates p<0.05.
(TIF)
