# Supplementary figures and images for: IVIg Immune Reconstitution Treatment Alleviates the State of Persistent Immune Activation and Suppressed CD4 T Cell Counts in CVID
Source: PLoS One. 2013 Oct 9;8(10):e75199. doi: 10.1371/journal.pone.0075199 (PMC3793979; doi:10.1371/journal.pone.0075199)

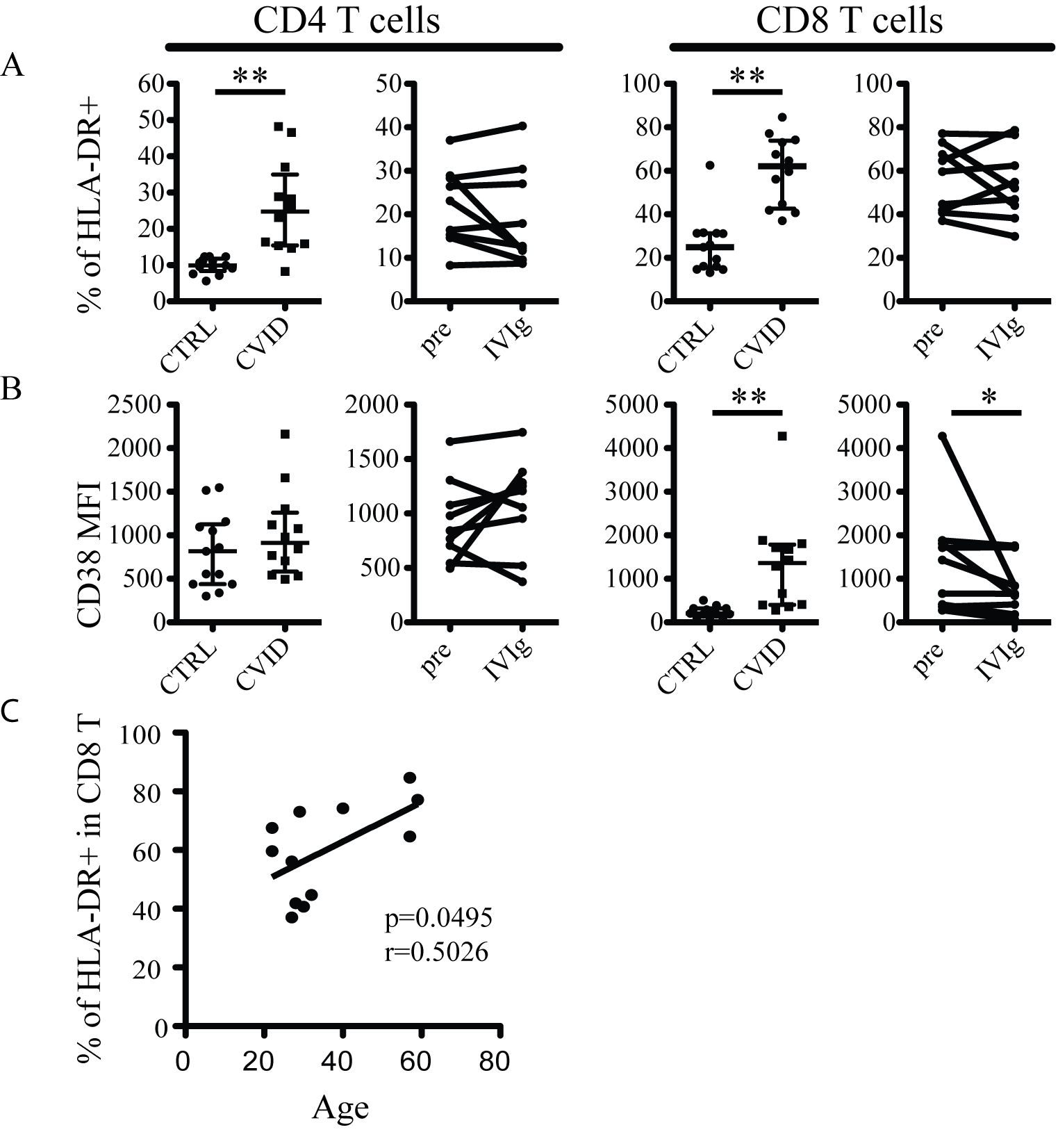

Supplement: Figure S1 — Immune activation in CD4 and CD8 T cells in CVID patients and the effect of immune reconstitution treatment. Comparison of the levels of HLA-DR+ cells (A), CD38 MFI (B), between healthy control and CVID patients (left panel), and in CVID patients before and after IVIg (right panel) for CD4 and CD8 T cells. (C) Correlation between age of the patients at baseline and the levels of HLA-DR expressing CD8 T cells. ** indicates p<0.002 and * indicates p<0.05. (TIF) [file pone.0075199.s001.tif]
